# Supplementary material for: Gene Structure, Expression and Function Analysis of MEF2 in the Pacific White Shrimp Litopenaeus vannamei
Source: Int J Mol Sci. 2023 Mar 18;24(6):5832. doi: 10.3390/ijms24065832 (PMC10051702; doi:10.3390/ijms24065832)
Supplement: Supplementary file 1 [file ijms-24-05832-s001.zip › Supplementary Materials-Table and Figures.pdf]

Supplementary Materials—Table and Figures

Table S1. Primers for this manuscript.

Primer Names	Sequence	Tm	PCR Product	
MEF2-YG-F	GGATCAAATCAGAACCCATCA	57℃	176bp	qRT-PCR
MEF2-YG-R	TAGATGGCCTGAGAGTGTATT			
18S-F	TATACGCTAGTGGAGCTGGAA	56℃	136bp	
18S-R	GGGGAGGTAGTGACGAAAAAT			
dsMEF2-F	TAATACGACTCACTATAGGG AATGGCTTACAGTCTCCCGA	60℃	592bp	primers for dsRNA synthesis template amplification
dsMEF2-R	TAATACGACTCACTATAGGG TGAGAGGGTTAAGCGAAATG			
dsEGFP-F	TAATACGACTCACTATAGGG CAGTGCTTCAGCCGCTACCC	60℃	289bp	
dsEGFP-R	TAATACGACTCACTATAGGG AGTTCACCTTGATGCCGTTCTT			
LVAN23936-F	TGTTTATCACCCTTGGCACCCTG	58℃	121bp	transcriptome validation
LVAN23936-R	CTCGGGAGCAGAAGATGAAGGAT			
LVAN06526-F	AGTGGTTGCCGGACTAATGG	58℃	138bp	
LVAN06526-R	CCTTGCGGTCCGTACTTCTT			
LVAN17308-F	GACGGGCAAGGAGAACAAGA	58℃	233bp	
LVAN17308-R	TAATGATGGTCTCGCGGTCG			
novel-2124-F	TAGGTGCGGTAAGACAAAGGTAT	58℃	181bp	
novel-2124-R	CCGCTGCAGAACTACAAGGGATA			
LVAN06763-F	AGAGAAGGTGCCAACACACA	58℃	148bp	
LVAN06763-R	GCCGTACAGCTTCTCGAACA			
LVAN2347-F	ACGGACAACGCTGAATCACT	58℃	143bp	
LVAN2347-R	AGGCAGTACCAGCAGAACAC			
LVAN23843-F	CTGCGGTGACGAGATGACTT	58℃	155bp	
LVAN23843-R	CAGCCTTCACGCTGATCTCT			
LVAN14378-F	GGATAGCATAACCGGAAGGAAAC	58℃	115bp	
LVAN14378-R	GAGAAGAGGCAAACACACGAAGG			
LVAN15846-F	CATGGAGGCCAAGGACTACGAC	60℃	242bp	
LVAN15846-R	TTGAATCTTCTGTTACAGGGGG			
novel-2155-F	TCCTCGATATTGCCGGCTTC	60℃	172bp	
novel-2155-R	TCCATGCCGAAGTCAACGAA			

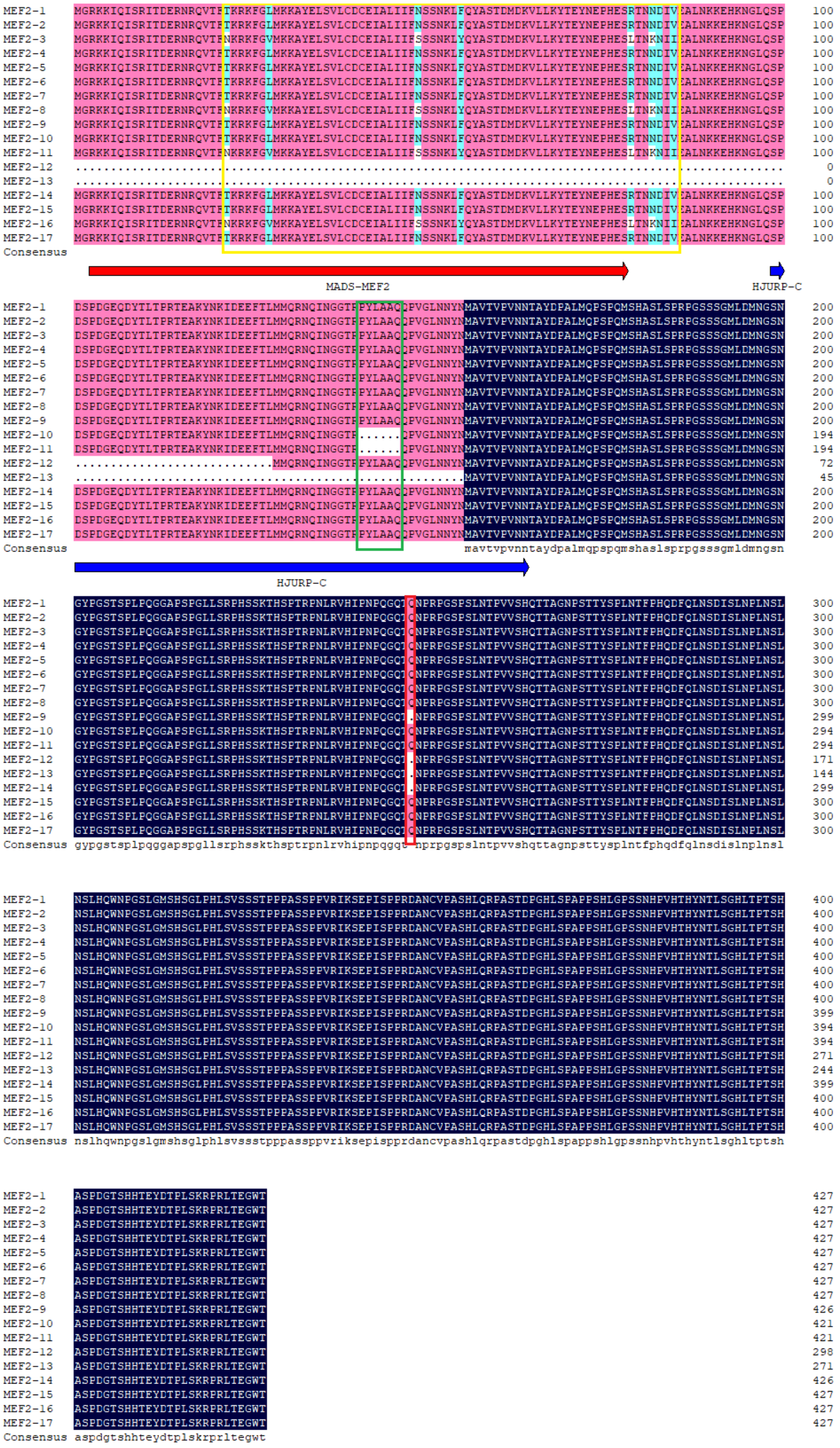

Figure S1. Multiple sequence alignment for the 17 protein sequences of *LvMEF2*. The yellow box corresponds to mutually exclusive exon 2. The green box corresponds to alternative 5′splice site of exon 4. The red box corresponds to alternative 5′splice site of exon 5. The red background corresponds to the functional domains. The black background corresponds to the C-terminal.

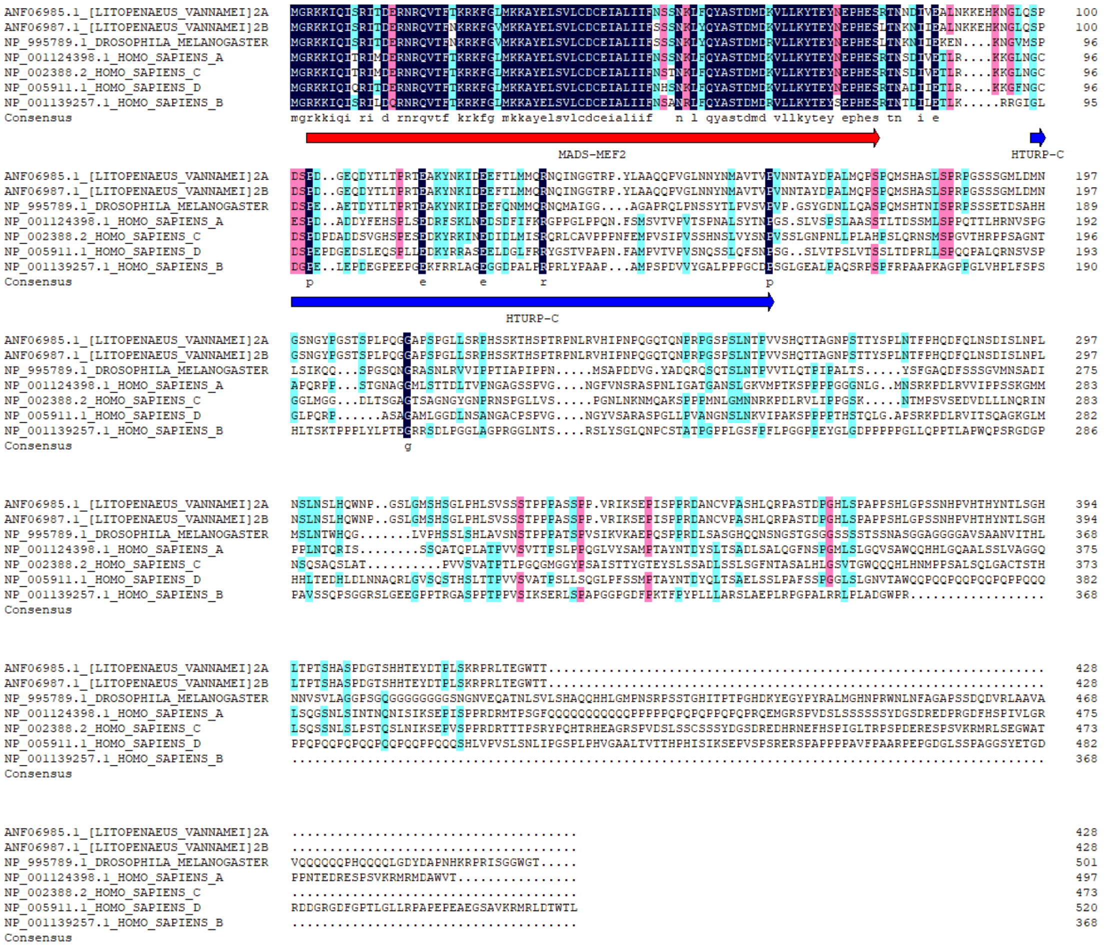

Figure S2. Multiple alignment of the MEF2 genes. The black, blue and red background correspond to the conserved sequences. The degrees of conserved are black>red>blue.

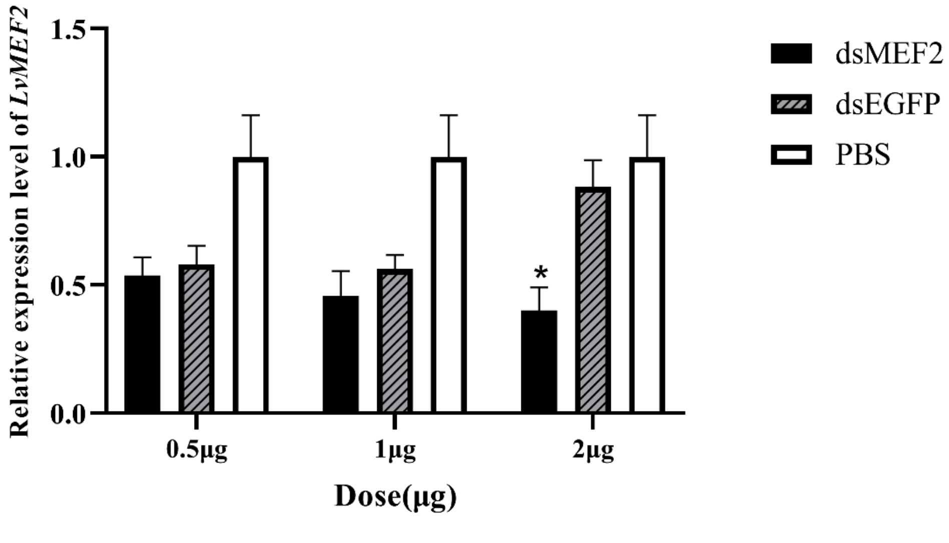

Figure S3. The optimal interference efficiency of *LvMEF2* after 48 hours of RNA interference. Significant differences of the gene expression levels between three treatments are shown as * $p < 0.05$.

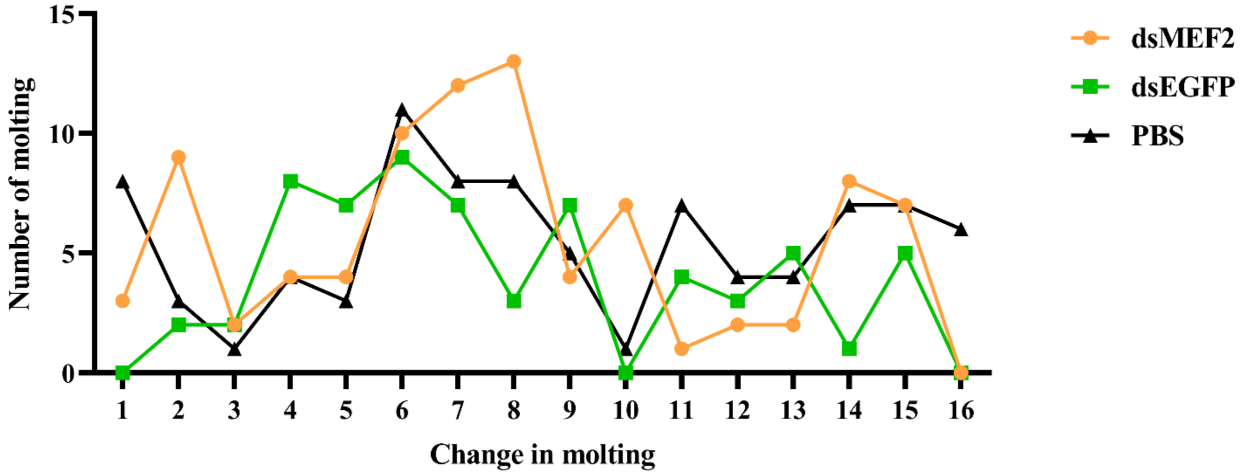

Figure S4. The molting situation during two weeks of RNA interference.

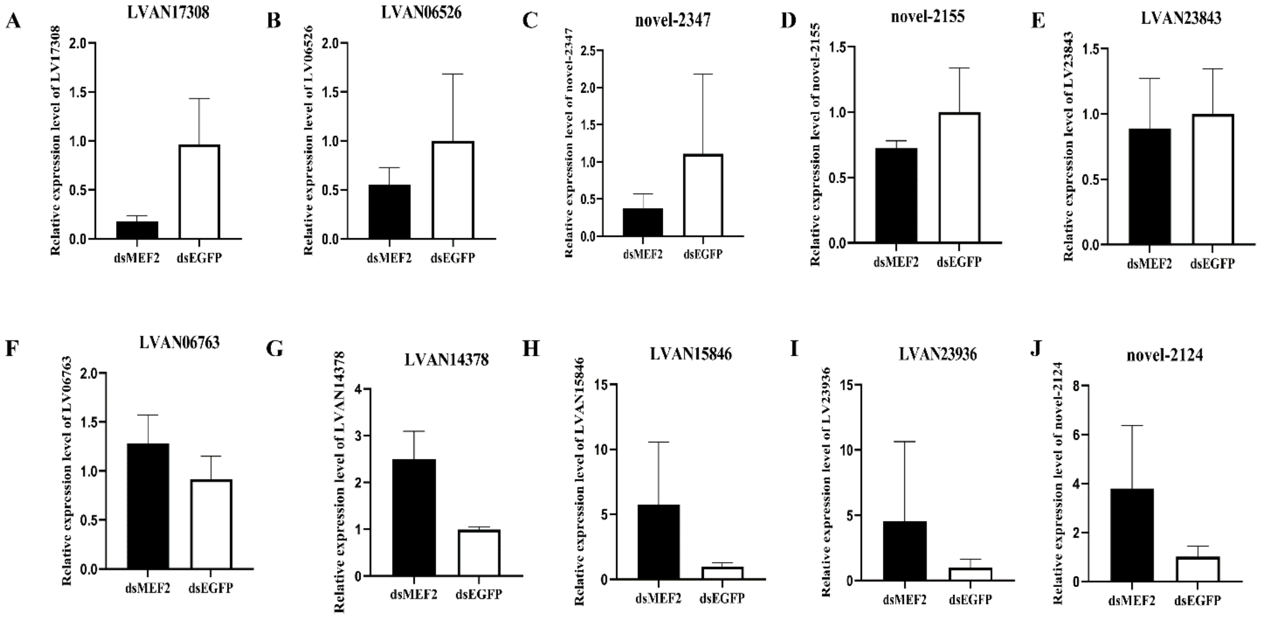

Figure S5. Expressions of related differential expression genes were verified after *LvMEF2* RNA interference. The gene annotations corresponding to the transcript numbers are as follows：A，LVAN17308 (Heat shock 70 kDa protein); B, LVAN06526 (Mitochondrial glycine transporter); C, novel2347 (E3 ubiquitin-protein ligase); D, novel-2155 (Myosin heavy chain, muscle); E, LVAN23843 (Heat shock protein 20); F, LVAN06763 (MAP kinase-interacting serine/threonine-protein kinase); G, LVAN14378 (Immunoglobulin I-set domain); H, LVAN15846(Glutaminase kidney isoform, mitochondrial); I, LVAN23936 (Myosin-4); J, novel-2124(Myosin heavy chain, muscle).
